# Insights Into the Assessment of WHO-Recommended Practices Based on Surgical Operations in Tertiary Healthcare Settings

**DOI:** 10.7759/cureus.83181

**Published:** 2025-04-29

**Authors:** Zeeshan Hussain, Asma Ambareen, Noor Ul Ain Rashid, Ahmad Raza, Sameer Anwar, Madeeha Minhas, Samreen Qureshi, Maryum Sana, Komal Zara

**Affiliations:** 1 Department of Underwater and Hyperbaric Medicine, Pakistan Navy Ship (PNS) Shifa Hospital, Karachi, PAK; 2 Department of Diving Medicine, Armed Forces Aero Medical Center, King Abdulaziz Air Base, Dhahran, SAU; 3 Department of Obstetrics and Gynecology, Khyber Teaching Hospital Peshawar, Peshawar, PAK; 4 Department of General Surgery, Akhtar Saeed Trust hospital, Lahore, Pakistan, Lahore, PAK; 5 Department of Surgery and Allied, Hayat Memorial Teaching Hospital, Lahore, PAK; 6 Department of Medicine, Continental Medical College, Lahore, PAK; 7 Department of Health Sciences, King Saud Bin Abdulaziz University for Health Sciences, Jeddah, SAU; 8 Department of Nursing and Patient Care, University of Health Sciences, Lahore, PAK; 9 Department of Pathology, University of Health Sciences, Lahore, PAK; 10 Department of Pathology, Federal Postgraduate Medical Institute, Lahore, PAK

**Keywords:** medical operations, surgery, surgical operations checklist, surgical safety checklist, who

## Abstract

Background: The protection of surgical safety comprises two major elements that produce the best outcomes for patients. The World Health Organization (WHO) created a surgical practice-based assessment for operative procedure-related risks. Medical professionals have proven the effectiveness of this checklist to eliminate both adverse outcomes and medical complications caused by surgical negligence.

Methodology: A study was conducted on 250 surgeries (major and minor) in a tertiary healthcare setting based on a qualitative questionnaire, adapted from the WHO checklist, operated through Google Forms. The examination spanned three months from September to November 2024. In accordance with WHO guidelines, the three surgical safety checklist phases - sign-in, time-out, and sign-out - were analyzed using SPSS version 20.0 (IBM Corp., Armonk, NY).

Results: The sign-out phase achieved the highest level of adherence, with 220 (88%) of surgical procedures using the checklist. The sign-in phase demonstrated 200 compliant cases (80%), whereas the time-out phase showed the lowest compliance, with only 170 cases (68%). Patient consent procedures, along with anesthesia protocols, instrument sterilization methods, and team member introduction protocols, all maintained complete success rates for ensuring a safe surgical space.

Conclusions: Implementing targeted awareness programs and training will help boost compliance rates with the WHO checklist, despite the current positive results.

## Introduction

A crucial aspect of health systems proves to be surgical care, but it brings unavoidable risks with it. Every year, thousands of millions of people receive surgical treatment, but numerous procedural complications affect their care-most of these complications have potential preventable causes [1-3]. Healthcare-related mortality rates combined with adverse patient outcomes occur more frequently in low- and middle-income countries because these territories encounter various limitations that impede safety protocols [4].

The World Health Organization (WHO) created the *Safe Surgery Saves Lives* initiative in 2007 to focus on the essential areas of anesthesia safety, infection control, and intraoperative communication [5]. The Surgical Safety Checklist (SSC) is the primary tool of their initiative, as it promotes surgical standardization and fosters team cohesion. Numerous studies after global implementation of the checklist confirmed its effectiveness by reducing surgical complications as well as death numbers, thus demonstrating its importance in all resource levels [6]. The SSC effectively boosts teamwork interactions within operating settings as well as physician responsibility measures [7,8].

The research determines the successful adoption rate of the SSC in a tertiary care hospital located in Lahore. The evaluation measures both the checklist implementation protocol and surgical outcome modifications after integration. The study focuses on identifying practical methods to reduce perioperative risks and enhance patient safety in clinics of a similar type.

## Materials and methods

A general assessment-based study was conducted from September 2024 to November 2024 in the tertiary healthcare setting via a convenient sampling technique. A total of 250 major and minor surgical procedures were included in the study. All major surgical patients were selected for enrollment, except those undergoing minimal procedures under local anesthesia.

The WHO's SSC transformed a closed-ended questionnaire with a checklist-based response format for assessment purposes through Google Forms. All data were obtained during surgical procedures through field observations, combined with SSC entries reviewed from patient medical records via an assessment form in Google Docs.

The general assessment-based study received study certification from the Postgraduate Medical Institute, Lahore (SURG331-23). The data analysis used SPSS version 20.0 (IBM Corp., Armonk, NY). There were three key stages: before anesthesia induction, before surgical incisions, and just before patient transfer to the recovery room (Table 1).

**Table 1:** WHO-recommended practices. According to WHO standards, perfect compliance is defined as complete adherence to every component of the checklist [9]. The study assesses compliance based on WHO guidelines across all three phases and presents the results in the table. WHO, World Health Organization

Sr. no.	Standards	Target	Evidence	Data source	Exception
Part I: Before Induction of Anesthesia					
1	Confirm the patient's identity, procedure, and consent	100%	WHO guideline	Direct observation/interview	None
2	Mark the surgical site	100%	WHO guideline	Direct observation/interview	None
3	Check the anesthesia machine and medications	100%	WHO guideline	Direct observation/interview	None
4	Known allergy	100%	WHO guideline	Direct observation/interview	None
5	Difficult airway/aspiration	100%	WHO guideline	Direct observation/interview	None
6	Risk of bleeding > 500 ml (7 ml/kg in children)	100%	WHO guideline	Direct observation/interview	Minor procedures with a low risk of bleeding
Part II: Before Start of Surgical Incision					
7	All team members introduce themselves by name and role	100%	WHO guideline	Direct observation/interview	None
8	Surgeon, anesthetist, and registered practitioner confirm patient name, planned procedure, site, and position	100%	WHO guideline	Direct observation/interview	None
9	Critical/unanticipated steps the surgeon may announce to the team	100%	WHO guideline	Direct observation/interview	None
10	Patient-specific concerns for the anesthetist	100%	WHO guideline	Direct observation/interview	None
11	Nurse confirms sterility of instrumentation	100%	WHO guideline	Direct observation/interview	None
12	Antibiotic prophylaxis within the last 60 minutes	100%	WHO guideline	Direct observation/interview	Not applicable if no prophylaxis is indicated
13	Essential imaging displayed	100%	WHO guideline	Direct observation/interview	Not applicable if no imaging required
Part III: Before Any Member of the Team Leaves the Operating Room					
14	The nurse verbally confirms the name of the procedure	100%	WHO guideline	Direct observation/interview	None
15	Confirm instruments, swabs, and sharps counts are complete	100%	WHO guideline	Direct observation/interview	Emergencies where counting is not feasible
16	Specimens labeled by patient name	100%	WHO guideline	Direct observation/interview	No specimen collected
17	Address any equipment problems	100%	WHO guideline	Direct observation/interview	If all equipment is functional
18	Report key concerns for recovery room professionals	100%	WHO guideline	Direct observation/interview	None

## Results

A total of 250 major surgical procedures were evaluated for WHO SSC compliance across three stages: sign-in, time-out, and sign-out. The sign-in phase achieved 200 (80%) compliance, during which healthcare teams confirmed patient identity, verified the procedure, and obtained consent. Staff also evaluated the functionality of anesthesia equipment, pulse oximetry devices, and medication supply systems. Patient allergies were documentation in 128 out of 250 (51.2%) surgeries, yet 122 (48.8%) procedures lacked known allergy records before surgical procedures. The low number of 105 surgical site markings in applicable cases indicates that this surgical practice needs to be strengthened. Risk documentation regarding significant blood loss existed in 145 (58%) of charted cases, but failed to show enough concern regarding identifying high-risk surgical situations.

The team member introduction procedure was recorded in 233 (93.2%) surgical procedures during the time-out phase. Anticipated blood loss, along with unanticipated steps and procedure duration, was communication in 245 (98%) instances among all cases. A lack of antibiotic administration for infection prevention existed in 73 (29.2%) of patients during surgeries because 177 (70.8%) healthcare providers correctly followed this procedure. Therefore, 73 patients remained at risk for developing postoperative infections. The results show insufficient preparation before surgery because the imaging display compliance was found at 117 (46.8%).

The sign-out phase exhibited the highest compliance rate because 220 surgeries (88%) followed the checklist procedures. Medical professionals documented the verification process that included checking procedure names and instrument completion status, together with sponge and needle counts, as well as reporting recovery room concerns. The moderate level of compliance in specimen labeling affected 183 procedures out of 250 (73.2%), yet manager check-ups received better compliance at 214 out of 250 (85.6%), indicating further improvements needed for complete adherence. The sign-out phase emerged as the most adhered-to level with 220 cases (88%), whereas time-out received the least compliance with 170 cases (68%). The sign-in phase demonstrated 200 (80%) cases of adherence during the assessment. The results are summarized in Table 2.

**Table 2:** The assessment outputs are documented in a filled WHO Scheme report. WHO, World Health Organization

Standards	Achieved	%	Skipped	%
Part I: Sign-in				
Confirm the patient’s identity, procedure, and consent	200	80%	50	20%
Mark the surgical site	105	42%	145	58%
Anesthesia machine and medication check	200	80%	50	20%
Pulse oximeter on the patient and functioning	200	80%	50	20%
Known allergy	128	51.2%	122	48.8%
Difficult airway or aspiration risk	145	58%	105	42%
Risk of >500 mL blood loss (7 mL/kg in children)	119	47.6%	131	52.4%
Part II: Time-out				
All team members introduce themselves by name and role	233	93.2%	17	6.8%
Surgeon, anesthetist, and nurse confirm verbally the patient's name, procedure, and site of incision	200	80%	50	20%
Antibiotic prophylaxis within the last 60 minutes	177	70.8%	73	29.2%
Critical/unanticipated steps	245	98%	5	2%
How long will the case take	245	98%	5	2%
Anticipated blood loss	245	98%	5	2%
Patient-specific concern for the anesthetist	245	98%	5	2%
Nurse confirmation about the sterility of instrumentation	200	80%	50	20%
Nurse confirmation about equipment issues or any concerns	156	62.4%	94	37.6%
Essential imaging displayed	117	46.8%	133	53.2%
Part III: Sign-out				
The name of the procedure	199	88%	30	12%
Completion of instruments, sponge, and needle counts	218	88%	30	12%
Specimen labeling (read specimen labels aloud, including patient name)	183	73.2%	67	26.8%
Whether there are any equipment problems to be addressed	214	85.6%	36	14.4%
Report key concerns for the recovery room professionals	220	88%	30	12%

## Discussion

The WHO SSC consists of three fundamental phases that match different points throughout surgical operations. The study matched previous research findings that the practice of verifying patient data achieved full compliance with international standards [10]. The operation team paid close attention to maintaining compliance with the hundreds of safety checks related to anesthesia administration. Time-out procedures were kept to protocol, but practitioners implemented them less frequently when compared to the sign-in and sign-out steps. The direct introduction of surgical personnel through both name and role achieved superior patient compliance compared to similar studies conducted within different health settings [11]. The improper timing of antibiotic prophylaxis administration among patients potentially raises their infection risk because antibiotics were administered outside their recommended period. The non-review of important imaging in this stage represented a critical mistake because medical imagery is vital for surgical decision-making operations and protecting patient safety [12]. 

The sign-out phase showed the greatest compliance because nurses perform it before moving patients to the recovery room. The sign-out phase ensures certain both surgical equipment is correctly accounted for and potential recovery room matters are properly managed. During the sign-out phase, the nurse double-checks the operation procedure name and inspects the presence of all surgical instruments while confirming specimen labels [13,14]. A high commitment to surgical safety became evident through complete compliance with established checkpoints during this phase [15]. Specimen labeling requires additional focus because it functions as a vital mechanism to associate a patient with their correct procedure diagnosis. The aspect generally receives minimal attention when staff shortages exist in multiple healthcare establishments [16]. The tables indicate that a minor delay occurred with report handovers (Table 2). The sign-out phase achieved the highest level of compliance since it reflected the dedicated commitment of nursing staff to protect patient safety.

The conducted research included various restrictions that could limit the transferability of its detected results. The short duration of the audit assessment reduced the ability to evaluate long-term SSC usage across different surgical teams during multiple shift times. Antibiotic prophylaxis timing and essential imaging review failed to be consistently performed, although both play important roles in patient safety, according to the findings. This research contains multiple advantages, together with various constraints. The surgical departments exhibit a high commitment to safety guideline practice as a main organizational strength. The operating room teams showed clear teamwork and effective speaking up behavior as members of the para-medical staff carried out their job responsibilities. Both the brief duration of auditing and the restricted participant number restrict how broad the available findings can be.

## Conclusions

Healthcare checklists, especially the SSC, demonstrate enormous value in enhancing healthcare outcomes. The evidence showed that surgical outcomes have improved through checklist usage, but surgeons encounter difficulties incorporating the checklist properly during operations. This research revealed strong implementation of WHO SSC procedures, which corresponds to fewer surgical complications.

The current compliance level stands below the optimal standard; thus, risks continue to exist. The need for heightened awareness about checklist compliance must be established to achieve better surgical safety results. Organizations should create reward programs for teams that demonstrate constant adherence to safety rules as a method to boost compliance.
